# Peritoneal adhesion index (PAI): proposal of a score for the “ignored iceberg” of medicine and surgery

**DOI:** 10.1186/1749-7922-8-6

**Published:** 2013-01-31

**Authors:** Federico Coccolini, Luca Ansaloni, Roberto Manfredi, Luca Campanati, Elia Poiasina, Paolo Bertoli, Michela Giulii Capponi, Massimo Sartelli, Salomone Di Saverio, Michele Cucchi, Daniel Lazzareschi, Michele Pisano, Fausto Catena

**Affiliations:** 1 General and Emergency Surgery department, Papa Giovanni XXIII hospital, Piazza OMS-Organizzazione Mondiale della Sanità 1, 24128, Bergamo, Italy; 2 General and Emergency Surgery department, Ospedale Maggiore, Parma, Italy; 3 General Surgery department, Macerata Hospital, Macerata, Italy; 4 General and Trauma Surgery department, Maggiore Hospital, Bologna, Italy

**Keywords:** Adhesions, Classification, PAI, Peritoneal, Abdominal, Occlusion, Surgery, Treatment, Prevention

## Abstract

Peritoneal adhesions describe a condition in which pathological bonds form between the omentum, the small and large bowels, the abdominal wall, and other intra-abdominal organs. Different classification systems have been proposed, but they do not resolve the underlying problem of ambiguity in the quantification and definition of adhesions. We therefore propose a standardized classification system of adhesions to universalize their definition based on the macroscopic appearance of adhesions and their diffusion to different regions of the abdomen. By scoring with these criteria, the peritoneal adhesion index (PAI) can range from 0 to 30, unambiguously specifying precise adhesion scenarios. The standardized classification and quantification of adhesions would enable different studies to more meaningfully integrate their results, thereby facilitating a more comprehensive approach to the treatment and management of this pathology.

## Article

Peritoneal adhesions are pathological bonds that typically form between the omentum, the small and large bowels, the abdominal wall, and other intra-abdominal organs. These bonds may be a thin film of connective tissue, a thick fibrous bridge containing blood vessels and nerve tissue, or a direct adhesion between two organ surfaces [1-3].

Depending on the etiology, peritoneal adhesions may be classified as congenital or acquired (post-inflammatory or post-operative) [4]. Some researchers assert that adhesions could also be classified in three major groups: adhesions formed at operative sites, adhesions formed de novo at non-operative sites, and adhesions formed after the lysis of previous adhesions [5]. Diamond et al. distinguished types 1 and 2 of postoperative peritoneal adhesions. Type 1, or de novo adhesion formation, involves adhesions formed at sites that did not have previous adhesions, including Type 1A (no previous operative procedure at the site of adhesion) and Type 1B (previous operative procedures at the site of adhesion). Type 2 involves adhesion reformation, with two separate subtypes: Type 2A (no operative procedure other than adhesiolysis at the site of adhesion) and Type 2B (other operative procedures at the site of adhesions) [6]. In 1990, Zhulke et al. proposed a classification of adhesions based on their macroscopic appearance, which has since been used expressly for experimental purposes [7]. These different classifications have no impact on the underlying problem of post-operative/post-inflammatory adhesions, which can be dramatic. Moreover these classification systems do not engender an unequivocal system of quantification and definition. Each surgeon defines adhesions on an individual basis contingent on the surgeon’s own experience and capability. At present, it is not possible to analytically standardize adhesions, even if such cases are a surgeon’s primary focus. The prevalence of adhesions following major abdominal procedures has been evaluated to be 63%-97% [8-12]. Laparoscopic procedures compared to open surgery have not demonstrated to significantly reduce the total number of post-operative adhesions [13-17]. Adhesions are a major source of morbidity and are the most common cause of intestinal obstruction [18,19], secondary female infertility, and ectopic gestation [20,21]. They may also cause chronic abdominal and pelvic pain [3,22,23]. Adhesive small bowel obstruction is the most serious consequence of intra-abdominal adhesions. Colorectal surgery has proven to be the most common surgical cause of intra-abdominal adhesions. Among open gynecological procedures, ovarian surgery was associated with the highest rate of readmission due to subsequent adhesions (7.5/100 initial operations) [24]. Retrospective studies have shown that 32%-85% of patients who require secondary abdominal surgery have adhesion-related intestinal obstruction. Experimental and clinical studies are not in agreement regarding the different rates of adhesion reformation following adhesiolysis performed via laparotomy or laparoscopy [25-27]. Guidelines have been published regarding the management of adhesive small bowel obstruction by the World Society of Emergency Surgery (WSES) [28].

Adhesions require highly involved surgical intervention and are a significant burden to health care systems. In the United States, an epidemiological study demonstrated that in 1988, 282,000 hospital admissions were attributable to adhesion-related disorders, and the cost of in-patient adhesiolysis procedures reached $1.18 billion [29]. Another study published in 1994, reported that 1% of all admissions in the United States involved adhesiolysis, costing $1.33 billion [30]. Adhesions and their associated complications have piqued both medical and legal interest in recent years [31]. Successful medical/legal claims include cases of bowel perforation following laparoscopic resolution of adhesion, delays in the diagnosis of adhesion obstruction of the small bowel, infertility resulting from adhesions, and visceral pain [31,32]. Currently, there is no effective method for preventing adhesion formation or reformation [33]. A more comprehensive understanding of the pathogenesis of adhesion formation at cellular and molecular levels is needed to streamline preventative treatment strategies [10].

The pathogenesis of adhesion formation involves three important trauma-induced processes: (I) inhibition of the fibrinolytic and extracellular matrix degradation systems [34,35]; (II) induction of an inflammatory response involving the production of cytokines and growth factor-β (TGF-β1), a key regulator of tissue fibrosis [36-38]; and (III) induction of tissue hypoxia following interruption of blood delivery to mesothelial cells and sub-mesothelial fibroblasts, leading to increased expression of hypoxia-induced factor-1α [39,40] and vascular endothelial growth factor, responsible for collagen formation and angiogenesis [31,41].

Several trials have examined the effects of systemic and local application of a variety of drugs, including steroids [41,42], non-selective and selective cyclooxygenase inhibitors [43-47], heparin [48-50], 3-hydroxy-3-methyl-glutaryl-CoA reductase inhibitors (statins) [51], and tissue-plasminogen activator [52]. Different theoretical approaches involving, for example, growth factors or the neurokinin-1 receptor, have also been tested. Further, the use of natural agents such as pollen and honey or cold saline solutions has been explored in an effort to reduce adhesion rates [53,54].

Local molecular therapies, including recombinant antibodies and protein, have been employed with moderate success [31]; these therapeutic agents work by correcting aberrant molecular pathways involved in adhesion formation [31]. Local molecular therapy is inherently limited; therefore an alternative strategy using gene therapy has recently been employed to correct molecular aberrations induced by surgical trauma [31]. In five studies based on rat models, different vectors were used to express therapeutic nucleic acids (transgenes or small interfering RNAs) in peritoneal tissue [31,40,55-59].

However, no method has distinguished itself as the optimal means of preventing adhesion formation [59]. Current preventive approaches range from the use of physical barriers to the administration of pharmacological agents, recombinant proteins and antibodies, and gene therapy, yet they have all failed to consistently yield satisfactory results. Single therapeutic strategies are typically unsuccessful in preventing peritoneal adhesions due to the multi-factorial nature of adhesion pathogenesis. Extensive literature on the subject demonstrates both the complexity of the issue and the myriad resources allocated to this condition, yet few interdisciplinary studies have been conducted involving experts from different fields. At this time the medical community only recognizes the “tip of the iceberg” and will continue treating the condition inadequately until it is more comprehensively explored.

**Figure 1:**
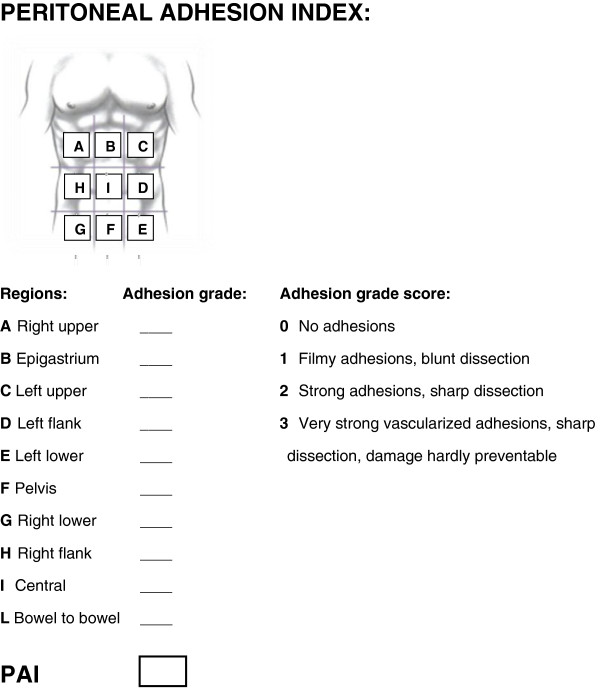
**Peritoneal adhesion index**: **by ascribing to each abdomen area an adhesion related score as indicated, the sum of the scores will result in the PAI.**

We are in agreement with Hellebrekers et al. and believe that additional prospective studies must be conducted to examine adhesion formation in relation to factors of inflammation, coagulation, and fibrinolysis. To more effectively integrate the findings of different studies, specific attention should be paid to uniformity of measurement (what, where, and when to measure) [60]. We therefore suggest a regimented classification system for adhesions in an effort to standardize their definition and subsequent analysis. In this way, different surgeons in different treatment centers can more effectively evaluate patients and compare their conditions to past evaluations using a universal classification system (Figure 1). This classification is based on the macroscopic appearance of adhesions and their extent to the different regions of the abdomen. Using specific scoring criteria, clinicians can assign a peritoneal adhesion index (PAI) ranging from 0 to 30, thereby giving a precise description of the intra-abdominal condition. Standardized classification and quantification of adhesions would enable researchers to integrate the results of different studies to more comprehensively approach the treatment and management of adhesion-related pathology.

Furthermore, as asserted by other researchers [53], we must encourage greater collaboration among basic, material, and clinical sciences. Surgery is progressively becoming more dependent on the findings of research in the basic sciences, and surgeons must contribute by practicing research routinely in a clinical setting. To further advance surgical techniques, we must better understand the physiopathology of surgically induced conditions.

## Competing interests

All authors declare to have no competing interests.

## Authors’ contribution

FCo, LA, FCa: Conception of the score, literature search and manuscript production. RM, LC, EP, PB, MS, SDS: literature search and analysis. MC, MGC, DL, MP: practical evaluation of the score. All authors read and approved the final manuscript.
